# Age-Related Inflammatory Balance Shift, Nasal Barrier Function, and Cerebro-Morphological Status in Healthy and Diseased Rodents

**DOI:** 10.3389/fnins.2021.700729

**Published:** 2021-07-23

**Authors:** Zsófia Varga-Medveczky, Noémi Kovács, Melinda E. Tóth, Miklós Sántha, Ildikó Horváth, Luca Anna Bors, Katalin Fónagy, Timea Imre, Pál Szabó, Domokos Máthé, Franciska Erdő

**Affiliations:** ^1^Faculty of Information Technology and Bionics, Pázmány Péter Catholic University, Budapest, Hungary; ^2^Department of Biophysics and Radiation Biology, Faculty of Medicine, Semmelweis University, Budapest, Hungary; ^3^Institute of Biochemistry, ELKH Biological Research Centre, Szeged, Hungary; ^4^Heart and Vascular Centre, Faculty of Medicine, Semmelweis University, Budapest, Hungary; ^5^Research Centre for Natural Sciences, Centre for Structural Study, Budapest, Hungary; ^6^Hungarian Center of Excellence for Molecular Medicine (HCEMM), Advanced In Vivo Imaging Core Faciltiy, Budapest, Hungary

**Keywords:** aging, cytokines, APOB-100 mice, APP-PSEN1 mice, magnetic resonance imaging, *in vivo* microdialysis, Alzheimer’s disease, nose-to-brain delivery

## Abstract

Increased blood–brain barrier (BBB) permeability and extensive neuronal changes have been described earlier in both healthy and pathological aging like apolipoprotein B-100 (APOB-100) and amyloid precursor protein (APP)–presenilin-1 (PSEN1) transgenic mouse models. APOB-100 hypertriglyceridemic model is a useful tool to study the link between cerebrovascular pathology and neurodegeneration, while APP–PSEN1 humanized mouse is a model of Alzheimer’s disease. The aim of the current study was to characterize the inflammatory changes in the brain with healthy aging and in neurodegeneration. Also, the cerebro-morphological and cognitive alterations have been investigated. The nose-to-brain delivery of a P-glycoprotein substrate model drug (quinidine) was monitored in the disease models and compared with the age-matched controls. Our results revealed an inflammatory balance shift in both the healthy aged and neurodegenerative models. In normal aging monocyte chemoattractant protein-1, stem cell factor and Rantes were highly upregulated indicating a stimulated leukocyte status. In APOB-100 mice, vascular endothelial growth factor (VEGF), platelet-derived growth factor (PDGF-BB), and interleukin-17A (IL-17A) were induced (vascular reaction), while in APP–PSEN1 mice resistin, IL-17A and GM-CSF were mostly upregulated. The nasal drug absorption was similar in the brain and blood indicating the molecular bypass of the BBB. The learning and memory tests showed no difference in the cognitive performance of healthy aged and young animals. Based on these results, it can be concluded that various markers of chronic inflammation are present in healthy aged and diseased animals. In APOB-100 mice, a cerebro-ventricular dilation can also be observed. For development of proper anti-aging and neuroprotective compounds, further studies focusing on the above inflammatory targets are suggested.

## Introduction

During the last years, several publications reported the effect of healthy aging on the permeability of the blood–brain barrier (BBB) (Erdő et al., 2017; Bors et al., 2018; Erdő and Krajcsi, 2019). Also, the efflux transporter downregulation has been documented in correlation with advanced age (Bors et al., 2018). The process of aging is in close connection with a kind of chronic inflammation and oxidative stress (Erdő et al., 2017). Similar observations have been published in chronic neurodegenerative disorders like Alzheimer’s disease, vascular dementia, and atherosclerosis (Erdő et al., 2017). Apolipoprotein B-100 (APOB-100) is the main structural protein of the triglyceride-rich very-low-density and cholesterol-enriched intermediate- and low-density lipoprotein (LDL) particles. Therefore, the overexpression of APOB-100 protein in mice leads to elevated plasma triglyceride level even on normal chow diet (Bereczki et al., 2008; Lénárt et al., 2012). Several studies demonstrated that increased serum LDL and APOB-100 levels in Alzheimer’s disease patients are associated with the pathological symptoms (Tóth et al., 2020). Indeed, APOB-100 overexpressing mice show many signs of neurodegeneration, such as synaptic dysfunctions, tau hyperphosphorylation, amyloid plaque formation (in homozygous mice), apoptosis, or the enlargement of the third and lateral ventricles in the brain (Bereczki et al., 2008; Lénárt et al., 2012). The chronic hypertriglyceridemia due to high serum APOB-100 level may lead to the functional and morphological changes of the BBB, which have also been described in this model (Hoyk et al., 2018). Therefore, APOB-100 overexpressing mouse strain is a useful model to study the age-related cerebrovascular pathology and neurodegeneration induced by hyperlipidemia, as the symptoms develop after 7–8 months of age (Tóth et al., 2020).

There are different transgenic models of Alzheimer’s disease. Since mouse models containing only presenilin (PSEN) mutated genes show an increased proportion of amyloid-beta (Aβ)42 but do not exhibit amyloid plaques, bigenic lines have been developed by crossing transgenic mice overexpressing the mutant form of amyloid precursor protein (APP) and PSEN1. Typically, these bigenic mice display an earlier onset and a more rapid rate of pathogenesis than monogenic lines, in both terms of amyloid accumulation and cognitive impairment (Esquerda-Canals et al., 2017).

Alzheimer’s disease pathophysiology entails chronic inflammation involving innate immune cells, namely, microglia, astrocytes, and other peripheral blood cells. Inflammatory mediators, such as cytokines and complements, are also linked to Alzheimer’s pathogenesis. Despite increasing evidence supporting the association between abnormal inflammation and Alzheimer’s disease, no well-established inflammatory biomarkers are currently available for the diagnosis. Since many reports have shown that abnormal chronic inflammation accompanies the disease, non-invasive and readily available peripheral inflammatory biomarkers should be considered as possible indicators for early diagnosis (Park et al., 2020). For human theranostics, mainly the peripheral plasma markers can be applied, but for determination of the most relevant factors and crucial mechanisms, the biomarkers characterized from brain homogenates in preclinical studies have also high importance.

It is widely accepted that atherosclerosis also involves chronic inflammation of blood vessel walls. Soeki and Sata (2016) reviewed the relationship between atherosclerosis and the dynamics of various inflammatory biomarkers, focusing on the development and progression of coronary artery diseases. The initial stages of atherosclerosis are often asymptomatic; however, when an atherosclerosis patient becomes symptomatic, his or her quality of life is significantly impaired. Therefore, early detection, diagnosis, and treatment of atherosclerosis is essential. Cytokines are a class of high molecular weight polypeptides that deliver cell signals in the context of immunological responses, inflammatory reactions, hematopoiesis, and other basic biological functions. For example, interleukin (IL)-6 and tumor necrosis factor (TNF)-α, members of the inflammatory cytokine family released from vascular smooth muscle cells, endothelial cells, monocytes, macrophages, and so forth, have been shown to be deeply involved in atherosclerosis. The evidence that links inflammatory markers to disease and prevention of disease is much greater for some inflammatory markers than for others. These markers provide valuable tools to study disease progression and new prevention strategies. Their value in clinical practice is still being investigated.

As for the molecular background of physiological healthy aging, a low-grade chronic inflammation is described to be present also in normal conditions of aging. The nuclear factor (NF)-κB signaling pathway has been recognized as the most important key process underlying this inflammation. Several studies reported that age-related NF-κB signaling upregulates the expression of the proinflammatory genes, TNF-α/β, ILs (IL-1β, IL-2, and IL-6), chemokines [IL-8; regulated on activation, normal T cell expressed and secreted (RANTES)], and adhesion molecules (AMs) (Chung et al., 2011). Furthermore, NF-κB-mediated upregulation of proinflammatory molecules, such as C-reactive protein (CRP), IL-6, and TNF-α, is closely associated with various age-related chronic pathophysiological conditions (Chung et al., 2006). The degree and kinetics of the upregulation correlate with the severity of the age-related clinical symptoms that can also be influenced by life style elements (like physical exercises, caloric restriction, and cognitive training).

In the current study, the models of healthy aging (in rats) and pathological aging (in APOB-100 and APP–PSEN1 mice) were studied to characterize the expression profile of inflammatory mediators in the brain, to analyze the nasal barrier permeability and function with aging, and also to study the possible morphological changes in the cerebral structures compared with healthy young or age-matched wild-type (WT) animals.

## Materials and Methods

### Animals

All animal experiments were performed in full compliance with the guidelines of the Association for Assessment and Accreditation of Laboratory Animal Care International’s expectations for animal use, in the spirit of the license issued by the Directorate for the Safety of the Food Chain and Animal Health, Budapest and Pest County Agricultural Administrative Authority, Hungary. The animals were kept at 22 ± 3°C and 50 ± 20% humidity animal room with a 12-h light/dark cycle and free access to food and water before and during the experiments.

#### Rats

Male young (2–3 months) and aged (14–21 months) Wistar rats (ToxiCoop Ltd., Budapest, Hungary) were compared in the behavioral studies, striatal protein assay, and brain cytokine ELISA plate array.

#### Mice

The APOB-100 transgenic mouse strain overexpressing the human APOB-100 protein was previously established by the group of Miklós Sántha (Bjelik et al., 2006), while B6C3-Tg(APPswe/PS1dE9)85Dbo/Mmjax mice were purchased from The Jackson Laboratory (Bar Harbor, ME, United States). Both mouse strains were maintained on a C57BL/6 genetic background in a hemizygous form. Breeding of the transgenic mouse strains were approved by the regional Station for Animal Health and Food Control (Csongrád County, Hungary; project licenses: XVI./2724/2017 for APOB-100 mouse strain and XVI./1248/2017 for APP/PS1 mouse strain). To determine the genotype of hemizygous transgenic animals and WT littermates, DNA from tail biopsies of pups was purified, and the presence of the transgenes was detected by PCR, as described earlier (Bjelik et al., 2006; Tóth et al., 2013).

For cytokine array and magnetic resonance imaging (MRI) studies, 8–11 months old male transgenics and WT mice were used. For microdialysis experiments, male APOB-100 mice and male and female APP–PSEN1 mice were used at the age of 8–11 months.

### Brain Homogenate Preparation

The rats were anesthetized [400 mg/kg chloral hydrate intraperitoneally (i.p.)] and decapitated, and the left striatum was quickly removed and weighed. Then, 1 ml of cold 1× cell lysis buffer, diluted from 2× cell lysis buffer for ELISA (EA-0001, Signosis Inc., Santa Clara, CA, United States) by Milli-Q water, was added to every 100 mg of tissue. The brain samples were then homogenized by tissue homogenizer (Ultra-Turrax TP 18/10; Staufen, Germany) on ice for a minute until the sample became entirely homogenous. The lysates were sonicated on ice for 30 s and then centrifuged at 10,000 rpm for 5 min at 4°C. The supernatants were collected and divided into aliquots. The aliquots were handled as quickly as possible to reduce the risk of protein degradation. Finally, the samples were stored in a freezer at −80°C until further analysis.

### Protein Assay

Pierce^TM^ BCA Protein Assay Kit (Thermo Fisher Scientific, Waltham, MA, United States) was used for protein determination. First, the albumin standards and the samples were prepared. The aliquots were diluted to one-tenth. The standards and the diluted samples were placed into a 96-well plate, and then the freshly mixed reagents were added. The plate was covered, gently shaken for 30 s, and placed into an incubator for 30 min at 37°C. After it cooled down to room temperature, the plate was placed into the plate reader (Tecan Spark 20M; Männedorf, Switzerland), and the absorbance was measured at 562 nm.

### Cytokine ELISA Plate Arrays

#### ELISA in Rat Samples

A 96-well chemiluminescence ELISA array from Signosis, Inc. (Rat Cytokine ELISA Plate Array, Catalog Number: EA-4004; Santa Clara, CA, United States) was used to detect cytokines in the striatal samples of aged and young rats. The assay was performed according to the description of the manufacturer. The brain homogenate supernatants were diluted to 100 μg/ml of total protein calculated from protein assay results.

**FIGURE 1:**
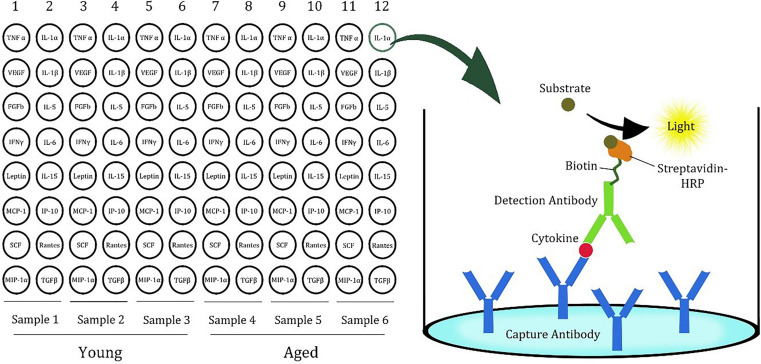
Plate design of cytokine ELISA array for rat samples. (Modified version of the picture provided by the manufacturer, Signosis).

A 96-well white plate was divided into six sections, and the sections included three samples. Three sections were used for three young rats as control, while the other three were used to measure the cytokines in the brains of the aged rats (Figure 1). In each section, the wells contained 16 specific cytokine capture antibodies. The cytokines in the test sample were sandwiched with first and second antibodies and visualized by avidin–biotin–horseradish peroxidase (HRP) binding using a luminescent substrate. The luminescence was detected by a Tecan Spark 20M (Männedorf, Switzerland) plate reader.

#### ELISA in Mouse Samples

For determination of cytokine expression in mouse brain homogenates, Signosis Mouse Cytokine ELISA Plate Array I (EA-4003; Signosis Inc., Santa Clara, CA, United States) was used. The assay was performed according to the instruction of the manufacturer. The brain homogenate supernatants were diluted to 1,000 μg/ml of total protein calculated from protein assay results. All together 24 different cytokines were determined, and the luminescence was compared with the WT mice [luminescence intensity ratio (LIR)]. APOB-100 mice and APP–PSEN1 mice were compared with the group of WT mice. For each strain, a pool of the left hemisphere (striatum) of five animals was used.

**FIGURE 2:**
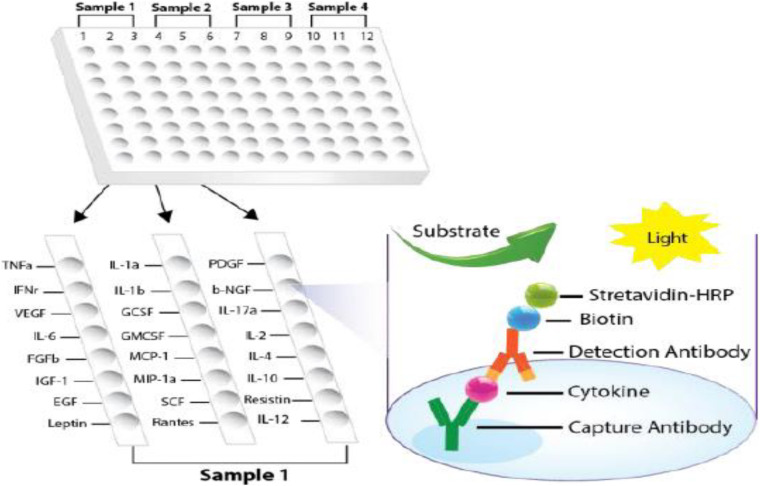
Plate design of cytokine ELISA array for mouse samples. (Modified version of the picture provided by the manufacturer, Signosis).

A 96-well white plate was divided into four sections, and the sections included two parallels for each sample. Two sections were used for the pool of five WT mice as control, while the other two were used to measure the cytokines in the brains of the pool of five transgenic mice (APOB-100 or APP–PSEN1, respectively). In each section, the wells contained 24 specific cytokine capture antibodies (Figure 2). The cytokines in the test sample were sandwiched with first and second antibodies and detected by avidin–biotin–HRP binding as a luminescent signal. The luminescence was detected similarly to rat assay.

### Morris Water Maze Test in Rats (a Pilot Study)

Morris water navigation task is supposed to measure spatial memory in rodents. The young and aged rats were trained for 4 days to escape onto a hidden platform from each of the cardinal starting positions (north, west, south, east) in the maze. The platform was placed in the south-east quadrant of the pool. Extra-maze cues in the lab were used to facilitate the orientation of the animals. The rats completed three daily trials with an intertrial interval of 30 min. Escape latency and swimming path were recorded using Smart v3.0 video tracking system software (Panlab, Spain). Escape latencies of the two groups were compared and analyzed by repeated measures ANOVA.

### Novel Object Recognition Test in Rats (a Pilot Study)

Novel Object Recognition (NOR) assay is a model for the investigation of recognition memory in rodents. The task procedure consists of two phases: Trial 1 (t_1_): familiarization with two identical objects in the test box; Trial 2 (t_2_): after 5 h intertrial delay, one of the familiar objects (O) was replaced by a novel object (N), and the exploration time of each object was measured for 3 min. The young and aged animals were observed through a video camera system. Recognition was characterized by the discrimination index (DI): (t_2*novel*_−t_2*fam*_)/(t_2*novel*_+t_2*fam*_) × 100; the higher the DI, the better recognition memory. DIs in the two groups were compared and analyzed with independent samples *t*-test.

### Magnetic Resonance Imaging

#### For Rats

T2-weighted anatomical scans were acquired on a 9.4T Varian (Agilent) Direct Drive MRI system (Varian Medical Systems Inc., Palo Alto, CA, United States) using a fast spin echo sequence (FSEMS) with the following parameters: repetition time (TR) = 5 s, echo train length (ETL) = 8, effective echo time (TE) = 36 ms, matrix = 128 × 128, field of view (FOV) = 22 mm × 22 mm, 30 slices with 1 mm thickness, and 2 averages.

#### For Mice

T2-weighted anatomical scans were acquired on a 1T preclinical nanoScan MRI scanner (Mediso Ltd., Budapest, Hungary) equipped with 450 mT/m gradients and a diameter of 20 mm transmit/receive volume coil. During the imaging, mice were anesthetized with 1.5% isoflurane in medical oxygen and placed in prone position on the MRI bed. A three-dimensional FSEMS was acquired with the following parameters: TR = 2 s, effective TE = 75.8 ms, ETL = 16, number of excitations = 3, matrix size = 120 × 96 × 64, and FOV = 30 mm × 30 mm × 19.2 mm.

Semi-automatic segmentation was used in VivoQuant software (inviCRO) to delineate the ventricles. First, a rough region of interest (ROI) was drawn on the brain manually, and then ventricles were segmented from it by connecting thresholding algorithm with thresholds calculated by Otsu’s method.

### *In vivo* Dual-Probe Microdialysis in Mice

#### Surgery and Sample Collection

Animals were anesthetized with chloral hydrate (450 mg/kg IP). The right jugular vein was exposed, and the MAB1.4.3 microdialysis probe was inserted into the vein. After checking the flow through the peripheral probe, the tubings of the probe were exteriorized under the scapulae. Then, the animals were placed in a Stoelting stereotaxic instrument, and the brain probe (MAB8.4.3) was implanted into the left striatum using the following coordinates with respect to the bregma: anterior–posterior (AP), +0.2 mm; from midline (ML), −2.2 mm; and dorso-ventral (DV), −3.2 mm. Microdialysis probes were connected to a CMA/102 microdialysis pump and perfused with artificial cerebrospinal fluid (aCSF, brain probe) or artificial peripheral perfusion fluid (aPPF, peripheral probe) at a flow rate of 1.0 μl/min. After a 30-min equilibration period, the animals were treated with intranasal (IN) QND to the left nostril, and then the sample collection was continued for 3 h. The microdialysate samples were collected every 30 min and placed on dry ice immediately. The frozen samples were stored at −80°C in a freezer until transferring them to the bioanalytical laboratory.

The IN administration volume for mice was 10 μl of 50 mg/ml for QND gel. For microdialysis experiments, aPPF (147 mmol/l NaCl, 4 mmol/l KCl, and 2.3 mmol/l CaCl_2_) and aCSF (147 mmol/l NaCl, 2.7 mmol/l KCl, 1.2 mmol/l CaCl_2_, and 0.5 mmol/l MgCl_2_) were prepared before the experiment using sterile Milli-Q water. All ingredients were purchased from Sigma-Aldrich (St. Louis, MO, United States).

#### Bioanalysis of Quinidine in Dialysate Samples

Identification of the quinidine (QND) concentration in the dialysate samples was performed on a Sciex 6500 QTrap hybrid tandem mass spectrometer coupled to an Agilent 1100 HPLC system. Electrospray ionization was used in positive ion detection mode with MRM transitions of 325.2/307.2 (quantifier) and 325.2/172 (qualifier) with a collision energy of 31 and 45 V, respectively. The dwell time of the transitions was 300 ms. Source conditions were: curtain gas: 45 arbitrary unit (au), spray voltage: 5,000 V, source temperature: 450°C, nebulizer gas: 40 au, drying gas: 40 au, and declustering potential: 171 V. The samples were introduced to the system *via* an HPLC system consisting of a binary pump, an autosampler, and a column compartment unit. A Phenomenex Synergi Fusion RP column (50 mm × 2 mm, 4 μm, 80 Å) column was applied for the separation using 0.1% formic acid in water as eluent A and 0.1% formic acid containing acetonitrile as eluent B in gradient elution mode. The gradient started at 90% of eluent A, and the eluent B was increased to 95% by 3 min and kept at that concentration for 0.5 min then decreased to the initial composition by 0.3 min and kept there for 2.2 min. The overall run time was 6 min. Then, 10 μl of samples was injected. The column was kept at ambient temperature. A 5-point calibration curve was used in the range of 0.1–100 ng/ml.

### Statistical Analysis of the Data

For rat ELISA cytokine plate array data, *in vivo* microdialysis results and NOR test independent samples Student’s *t*-test (by Microsoft Excel 2016) were used. For statistical analysis of the results of Morris water maze test, one-way ANOVA with repeated measures was applied. In the case of mouse ELISA plate array assay, pooled samples of five animals were used and tested in duplicates. In this assay and for the MRI data, only the mean values could have been calculated.

## Results

For proper preparation of striatal samples for cytokine ELISA array, a sufficient dilution of the supernatants was necessary. To make these solutions, first the total protein levels should have been determined. The results of protein assay for rats are shown in Table 1, and for mice, they are presented in Table 2.

**TABLE 1:** Age, body weight, and striatal total protein content in the supernatant for aged and young rats used for cytokine ELISA array studies.

Rat no.	Age of rats (month)	Body weight (g)	Weight of the homogenized striatum (mg)	Total protein (μg/ml)
1	2–3	250	174.0	5,474.6
2	2–3	252	173.1	5,441.5
3	2–3	285	207.8	5,091.2
4	16	530	266.4	4,949.1
5	21	588	206.8	4,927.2
6	21	510	180.3	4,869.9

**TABLE 2:** Age, body weight, and total protein content in the supernatant of the brain homogenates for WT and transgenic mice used for cytokine ELISA array studies.

Group of mice *n* = 5	Age (month) (mean ± SE)	Body weight (g) (mean ± SE)	Weight of the pool of homogenized hemispheres (mg)	Total protein (mg/ml)
Wild-type	10.5 ± 0.00	30.88 ± 1.86	155.6 ± 13.9	5,864.5
APO-B100	7.8 ± 2.08	31.90 ± 5.17	138.1 ± 9.0	5,577.0
APP–PSEN1	10.6 ± 0.22	31.11 ± 2.07	154.0 ± 15.2	6,383.5

### Total Protein Levels in the Rat Striatum

For determination of total protein level in the striatal supernatant, three old and three young rats were used. The results are shown in Table 1. The total protein values were higher in the young subjects than in the aged group. For cytokine ELISA plate array, the lysates were diluted to 100 μg/ml protein concentration.

In the case of mouse experiments for preparation of brain homogenates, a pooled sample of the left hemispheres of five animals/group was used. Otherwise, the total protein levels were determined similarly to rat experiments. The results are shown in Table 2. Afterward, the lysates were also diluted to reach 100 μg/ml protein concentration for cytokine assay.

### Cytokine Levels in Healthy Aged and Young Rats

**FIGURE 3:**
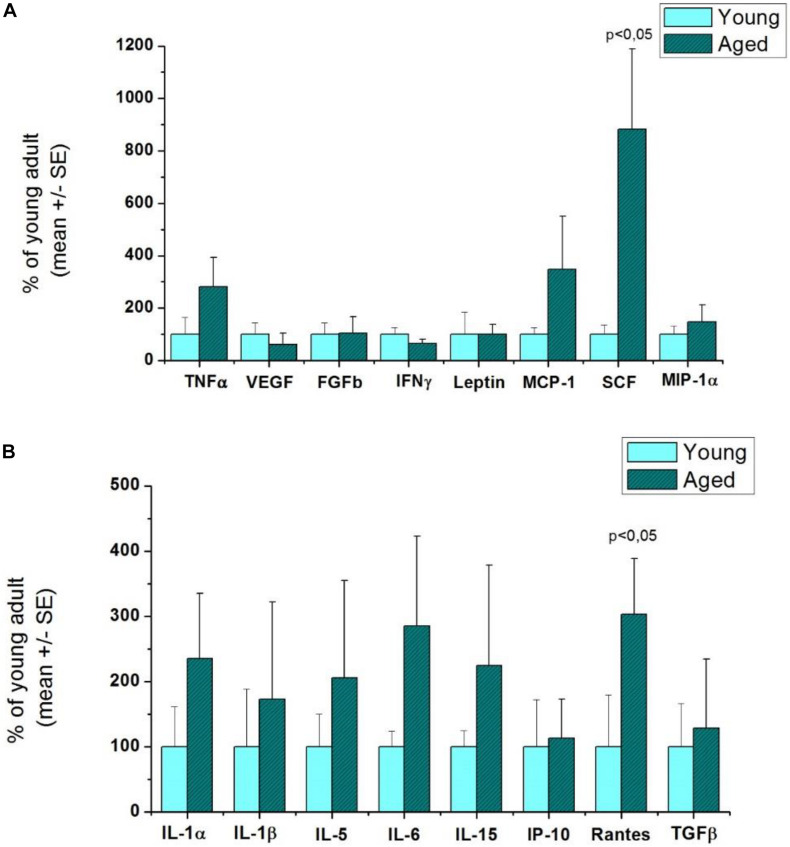
Comparison of inflammatory cytokine levels in aged and young rat striatums. *n* = 3/group. *p* < 0.05: statistically significant difference in the cytokine levels between the aged and young groups by Student’s *t*-test. **(A)** The first 8 cytokines in the plate. **(B)** The second 8 cytokines in the plate.

**FIGURE 4:**
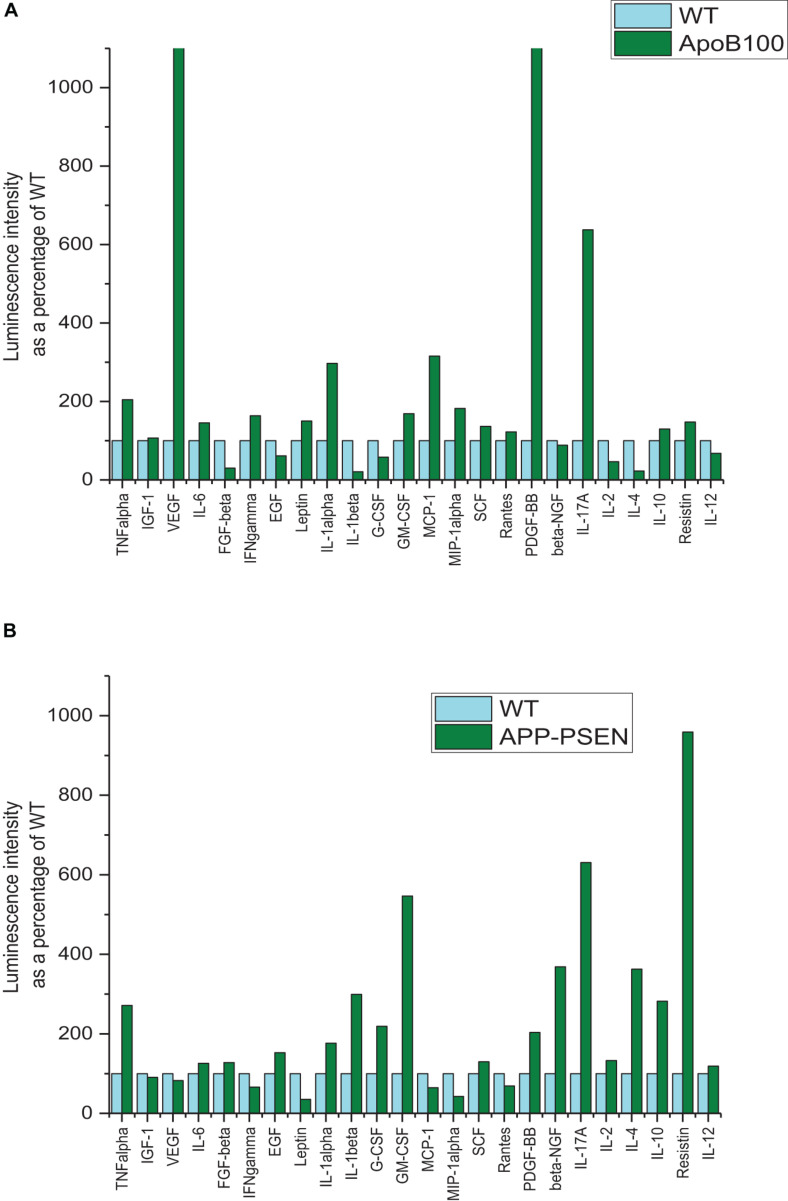
Comparison of cytokine expression in WT and transgenic mice. **(A)** APOB-100. **(B)** APP–PSEN1 mice. The samples were taken from the supernatant of pooled homogenates of the left hemisphere of a group of five animals. The measurements were performed in duplicates.

Cytokine profiling was performed for rat striatums by analyzing the expression of 16 cytokines (Figures 3A,B) and for mouse pooled hemispheres for 24 cytokines (Figures 4A,B).

In aged rats, stem cell factor (SCF) (8.82-fold increase), monocyte chemoattractant protein-1 (MCP-1) (3.48-fold increase), and Rantes (regulated on activation of normal T cells expressed and secreted) (3.03-fold increase) were the most upregulated cytokines compared with the young group (Figure 3). These results suggest pathological processes in the brain during healthy aging based on chemotaxis, leukocyte recruitment, and formation of new blood cells (hematopoiesis). It can also be observed in Figure 3 that the majority of inflammatory markers were also upregulated with healthy aging: IL-1α, IL-1β, IL-5, IL-15, and TNF-α.

The LIRs and the function of each cytokine of aged compared with young rats are shown in Table 3.

**TABLE 3:**
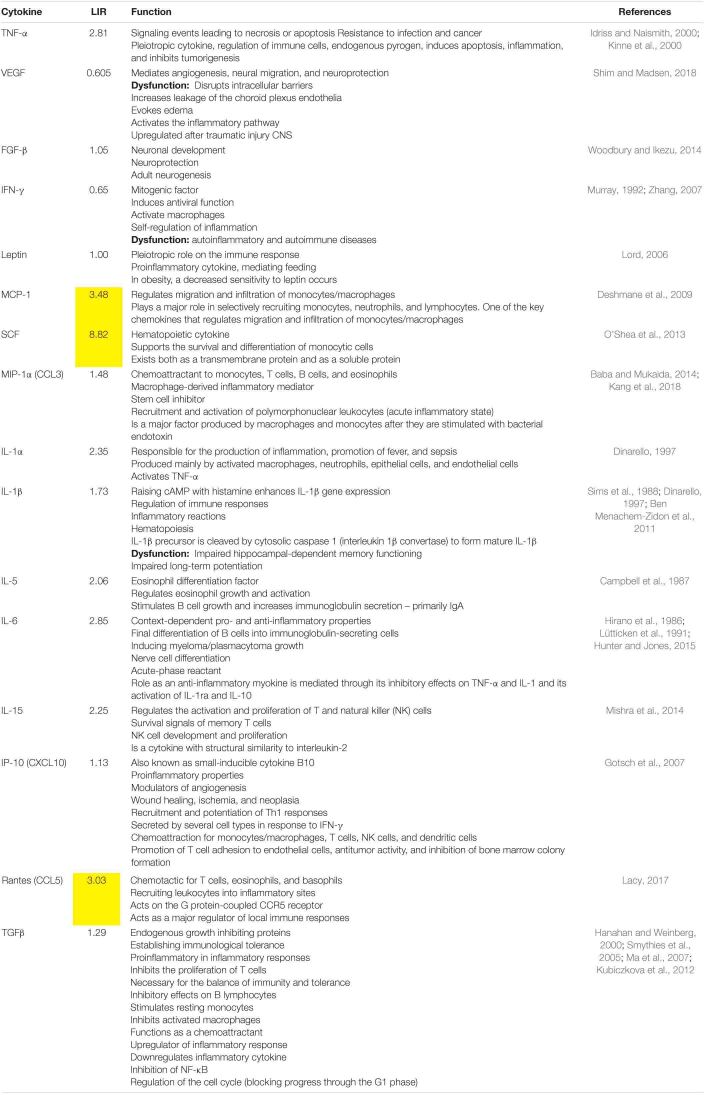
Biological function of the cytokines determined with cytokine ELISA plate array for rats.

*LIR, luminescence intensity ratio (aged/young). TNF-α, tumor necrosis factor-α; VEGF, vascular endothelial growth factor; FGF-β, basic fibroblast growth factor; IFN-γ, interferon γ; MCP-1, monocyte chemoattractant protein-1; SCF, stem cell factor; MIP-1α, macrophage inflammatory protein 1α; IL, interleukin; IP-10, interferon gamma-induced protein 10; Rantes, regulated on activation, normal T cell expressed and secreted; TGFβ, transforming growth factor β. Yellow highlighted values mean more than 3-fold upregulation contrary to young rats.*

### Cytokine Levels in WT and Transgenic (APOB-100 and APP–PSEN1) Mice

As it is known from the literature, APOB-100 transgenic mice are wildly accepted models of neurodegeneration with vascular origin (Bereczki et al., 2008; Hoyk et al., 2018). In this cytokine assay, vascular endothelial growth factor (VEGF) expression showed the highest increase, more than 13-fold upregulation than the control, in APOB-100 mice. VEGF is a signal protein produced by the cells stimulating blood vessel formation, and it induces angiogenesis in the brain. The second factor was platelet-derived growth factor (PDGF-BB) that also showed a dramatic upregulation (more than 11-fold than the WT) by the genetic modification of APOB-100 gene. Interleukin-17A (IL-17A), a proinflammatory cytokine produced by activated T cells, was also significantly increased by the genetic modification of APOB-100. This cytokine regulates the activity of NF-κB, and mitogen-activated protein kinase (MAPK) can stimulate cyclooxygenase-2 (COX-2) and enhance nitric oxide (NO) production. On the contrary, the expression of some other inflammatory factors like IL-1α, IL-1β, IL-2, and IL-4 was unchanged or downregulated in APO-B100 mice. These cytokines have a crucial role in inflammation and cellular immunity.

The double-humanized mouse model of Alzheimer’s disease (APP–PSEN1 mice) was also compared with littermate WT mice. The mostly upregulated cytokines in the brain were resistin (9.59-fold increase), IL-17A (6.31-fold increase), and granulocyte-macrophage colony-stimulating factor (GM-CSF) (5.47-fold increase). Resistin is secreted by adipose tissue and was shown to cause high level of bad cholesterol (LDL). Resistin accelerates the accumulation of LDL in the arteries, increasing the risk of vascular diseases. IL-17A is a proinflammatory protein, and GM-CSF regulates the macrophage number and function. It is a product of cells activated by inflammation and pathologic conditions.

The LIRs as a marker of relative expression levels of the 24 cytokines tested for APOB-100 and APP–PSEN1 mice are presented in Table 4. The comparative bar graphs are shown in Figures 4A,B.

**TABLE 4:**
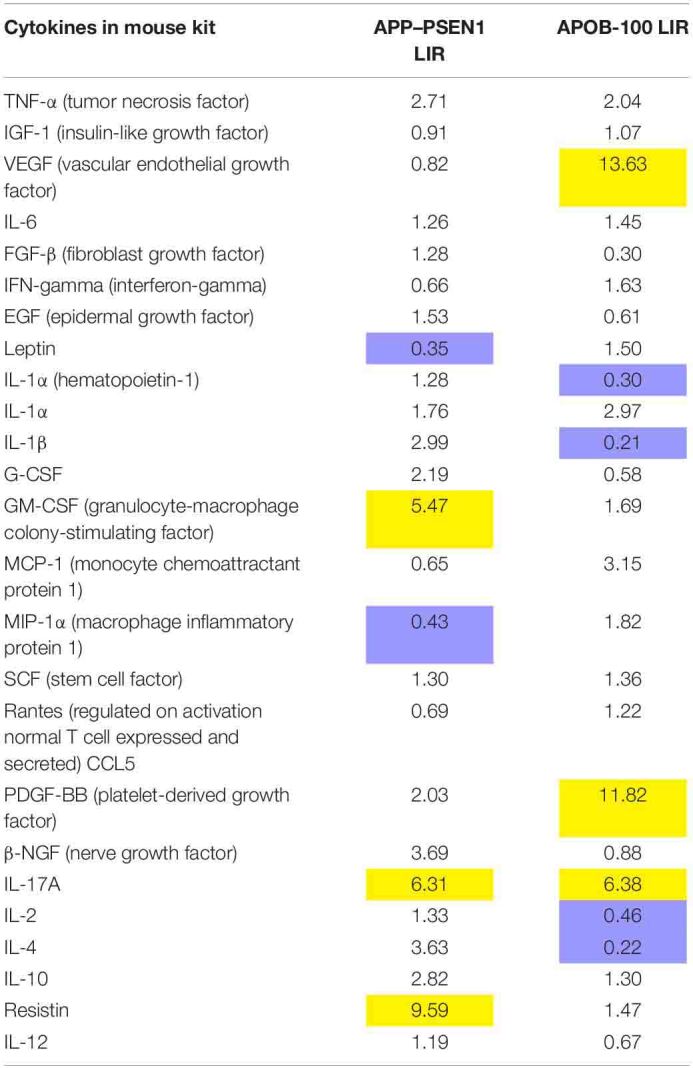
Luminescence intensity ratio (LIR) compared with WT mice.

*Yellow: more than 5-fold increase. Blue: less than 0.5-fold decrease in the luminescence intensity ratio compared with the littermate WT mice.*

### Morris Water Maze Test in Rats

**FIGURE 5:**
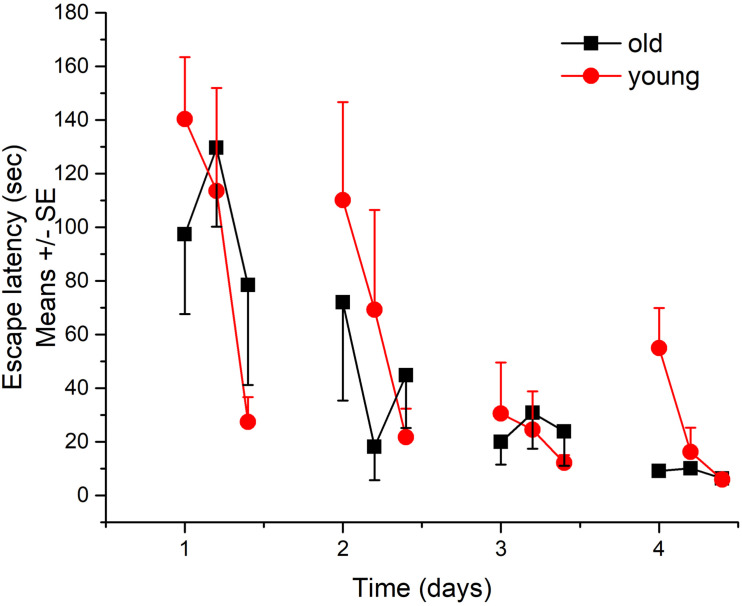
Learning and memory performance of young and aged Wistar rats in Morris water maze test. *N* = 4/group.

Both groups successfully learned the Morris test as shown by the significant decreases in the latency to find the hidden platform across 4 days (Figure 5). There was no significant difference between the performance of the two groups.

### NOR Test in Rats

Discrimination index: DI = (N−O)/(N+O)

**FIGURE 6:**
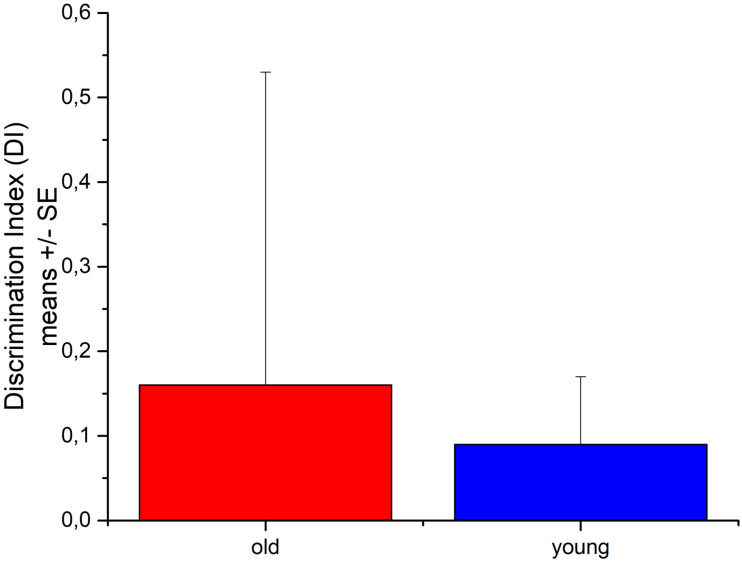
The recognition memory performance of young and aged Wistar rats in Novel Object Recognition test. *N* = 4/group.

Both old and young rats explored the novel and the familiar object on average about for the same time showing no sign of recognition memory (Figure 6). Three of the old rats were freezing in the test box during the majority of the t_2_ test period. Young rats explored the objects (novel + familiar) almost three times longer than old rats did; the difference just missed the 5% statistical significance.

### Brain MRI of Aged Rats

**FIGURE 7:**
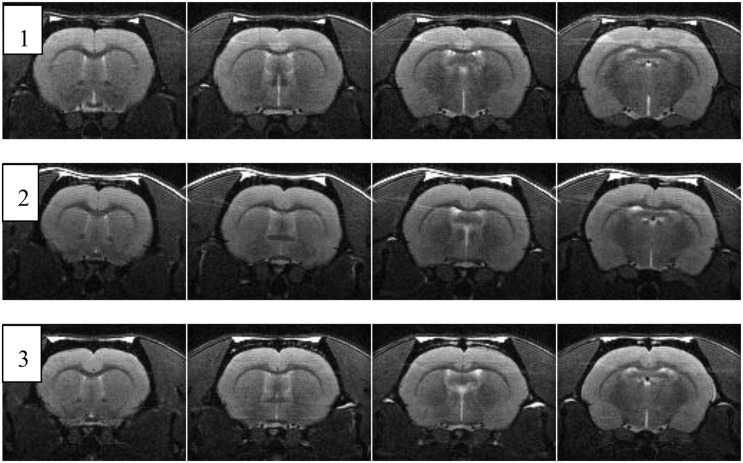
Four consecutive coronal MRI sections of the brain of three aged male Wistar rats [after (Bors L. et al., 2020), Corrigendum]. The age and body weights are shown in Table 5.

The results of MR imaging in old rats are presented in Table 5 and Figure 7. There were no significant morphological changes in the brains with advanced age.

**TABLE 5:** Age and body weight of the rats subjected to brain MRI.

Rat no.	Age of animals (months)	Body weight (g)
1	16	530
2	21	588
3	21	510

### Brain MRI of WT and Transgenic Mice

**FIGURE 8:**
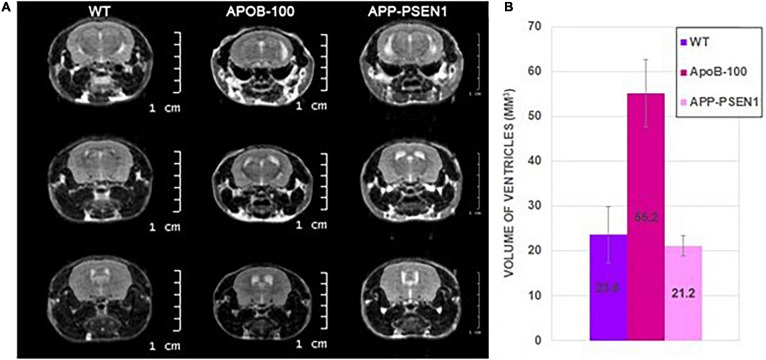
Representative coronal MR images of wild-type, APOB-100, and APP–PSEN1 mice with a special focus on the size of the cerebral ventricles **(A)**. Mean ventricle volumes of different mouse strains **(B)**.

Magnetic resonance imaging was acquired on three groups of mice: WT group of four male mice (301.8 ± 44 days old), APOB-100 group of four male mice (345.5 ± 5.5 days old), and APP–PSEN1 group of two male mice (398.5 ± 0.5 days old) (Table 6). The volumes of the segmented ventricles were determined for each mouse, and group means and standard deviations were calculated (Figure 8). APOB-100 mice had significantly enlarged ventricles (55.2 mm^3^) compared with WT and APP–PSEN1 mice (23.6 and 21.2 mm^3^, respectively).

**TABLE 6:** Age and body weight of the mice subjected to brain MRI.

Mouse	Age of animals (days/months)
WT 1	377/12.5
WT 2	365/12.2
WT 3	282/9.4
WT 4	283/9.4
APOB-100 1	351/11.7
APOB-100 2	351/11.7
APOB-100 3	340/11.3
APOB-100 4	340/11.3
APP–PSEN1	399/13.3
APP–PSEN2	398/13.3

### Brain Penetration of IN P-Glycoprotein Substrate QND in WT and Transgenic Mice

It is known from the literature that the IN delivery route of drug administration is able to bypass the BBB (Erdő et al., 2018). The nasally administered drugs can penetrate the brain *via* olfactory or trigeminal pathways (Erdő et al., 2018), and the molecules may cross the nasal mucosa also paracellularly (where the tight junctions are missing) or by the sensory neuronal endocytosis and reach the central nervous system (as the first intrusion places: the bulbus olfactorius and the brainstem) in a direct way. Then, the compounds are distributed in the entire brain parenchyma. In our experiments, QND, a well-known P-glycoprotein (P-gp) substrate (Sziráki et al., 2011), was applied intranasally in mice. In case of systemic administration, the brain penetration of this molecule is restricted by the P-gp efflux pump (Sziráki et al., 2011, 2013). Only approximately 30% of the blood level is reached in the brain after intravenous or intraperitoneal treatment (Sziráki et al., 2011, 2013) in rodents.

**FIGURE 9:**
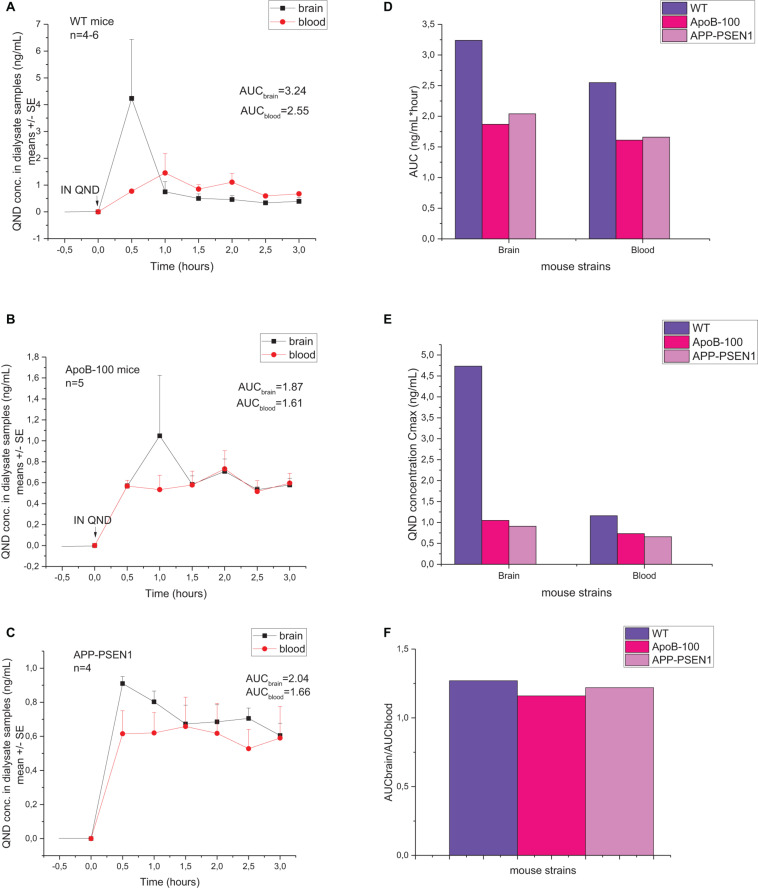
Concentration–time profiles of quinidine (QND) brain and blood penetration following intranasal administration in **(A)** wild-type, **(B)** APOB-100, and **(C)** APP–PSEN1 mice measured by dual-probe microdialysis. Comparison of area under the curve (AUC) values **(D)**, C_max_ values **(E)**, and AUC_brain_/AUC_blood_ **(F)** of wild-type and transgenic mice generated from brain and blood concentration–time curves of microdialysis results.

In the current experiments, QND is applied in a gelous vehicle, which ensures a continuous drug release and absorption during the observation period (3 h) (Figure 9). A previous experiment provided evidence that IN delivery in gel formulation has several advantages contrary to nasal solutions (Bors L.A. et al., 2020). In WT, APOB-100, and APP–PSEN1 transgenic mice, the nasal absorption pattern seems to be similar in our microdialysis experiments. After a rapid absorption peak, a long-lasting plateau phase is coming in the concentration–time profiles (Figures 9A–C). The C_max_ value and also the AUC value are the highest in the WT mice (Figures 9D,E), while the AUC_brain_/AUC_blood_ ratio is similar in all the three strains. The brain exposure is higher than the blood concentration in all the three groups of mice, suggesting no significant role of capillary endothelial efflux pumps in the nasal mucosa in the drug absorption.

Based on these dual-probe microdialysis results, it can be concluded that there is no remarkable difference in the nasal barrier function between the WT and diseased mice. Only a transiently higher brain uptake of QND can be seen in the early phase (0.5–1.0 h) after nasal exposure in the case of healthy animals compared with the APP–PSEN1 and APOB-100 mice.

## Conclusion and Discussion

This study aimed to analyze the process of healthy aging and also the age-related neurodegenerative diseases (Alzheimer’s disease, atherosclerosis) in rodents. The healthy aging was investigated in 14–21-month old rats, while the neurodegenerative processes were studied in APP–PSEN1 and APOB-100 transgenic mice in the age of 9–13 months when the disease symptoms have already been developed (Bjelik et al., 2006; Bereczki et al., 2008; Hoyk et al., 2018). The study focused on three main areas: the cerebral cytokine expression compared with healthy individuals; the anatomical and morphological changes in the brain using MRI compared with controls; and the examination of nasal barrier permeability for a P-gp substrate by *in vivo* dual-probe microdialysis. Also, the behavioral status was evaluated in rats using two memory and learning assays: Morris water maze test and NOR test.

Based on the results, it can be concluded that in normal aged rats, the role of the hematopoietic cytokine (SCF) is highly increased that may lead to the enhanced survival and differentiation of monocytic cells. Also, MCP-1 and Rantes are upregulated with advanced age, which processes are responsible for the growing cell migration and infiltration of monocytes/macrophages and chemotactic for T cells, eosinophils, and basophil leukocytes. All these processes lead to a chronic local inflammation and immune response in the brain in the old subjects. The immune-assay in mice showed also upregulated inflammatory markers. Enhanced VEGF and PDGF-BB levels were detected in APOB-100 transgenic mice. On the other hand, an early study revealed significantly lower microvascular density in the brain of APOB-100 transgenic animals than of WTs, suggesting a defective VEGF signaling (Süle et al., 2009). Indeed, it was demonstrated in APOE−/− mice that hyperlipidemia hindered VEGF-induced angiogenesis (Zechariah et al., 2013). Moreover, highly increased level of VEGF may induce the disruption of the BBB (Lange et al., 2016), which is in line with a previous result (Hoyk et al., 2018). In APP–PSEN1 mice, the increased level of resistin and GM-CSF was observed indicating the enhanced level of LDL and stimulated macrophage function. Previously, a significantly increased serum resistin level was found in human patients with Alzheimer’s disease (Demirci et al., 2017). Although resistin is secreted mainly by the adipocytes, it has been detected in other tissues and in the cerebrospinal fluid as well (Badoer et al., 2015). Moreover, the *in situ* production of resistin was proven in mouse brain (Morash et al., 2002). The proinflammatory cytokine, IL-17A, was upregulated in both diseased mouse strains, suggesting an overproduction of COX-2, IL-6, and NO. This cytokine also regulates NF-κB and MAPKs and is a marker of T cell activation.

Intranasal administration is a promising strategy to bypass BBB and deliver CNS dugs directly to the brain. However, the efficacy of this process can be influenced by different efflux transporters, such as P-gp. Altered function and expression of P-gp have been found in Alzheimer’s disease patients (van Assema et al., 2012) and in APOB-100 transgenic mice as well (Hoyk et al., 2018). Accordingly, investigating nasal barrier permeability in disease model animals is of primary importance for the future development of possible therapeutic methods. In nasal barrier studies, QND, a reference probe-substrate of P-gp was used for characterization of barrier permeability (Sziráki et al., 2011, 2013). In BBB, P-gp is the major efflux transporter responsible for the protection of the brain from xenobiotics. In the nasal cavity, there is a direct pathway of the molecules to be absorbed to the brain through the nasal mucosa bypassing the BBB. In the current experiment, the P-gp substrate was administered as a nasal gel formulation, and its penetration was monitored in the brain and in the periphery. In both diseased mouse strains and also the WT mice after a rapid absorption, a long-lasting continuous release and penetration of QND have been observed. These results indicate an unchanged nasal barrier function in the transgenic mouse models compared with WT and provide evidence that there is no remarkable role of BBB in drug absorption through the nose-to-brain axis in mice. On the contrary, a previous study described the role of peripheral P-gp transporters in the modulation of nasal drug to brain penetration in healthy rats (Bors L.A. et al., 2020).

It was hypothesized that the extensive neuronal death observed in APOB-100 and APP–PSEN1 mice should affect brain morphology. This was investigated using MRI in both strains and compared with the controls. Remarkable enlargement in the cavity size of the lateral and dorsal ventricles and a moderate increase in the aqueduct (fourth ventricle) size were detected in the brain of APOB-100 mice. On the other hand, no significant dilation of the ventricles was detected in the case of APP–PSEN1 mice compared with the WT. For APOB-100 mice, the dilation of the ventricles can be the consequence of enhanced production, infiltration, or defected drainage of the cerebrospinal fluid. The downregulation of the cerebral glymphatic in this strain with advanced age and the reduced function of the mitochondria (Bereczki et al., 2008) can also contribute to the lower pumping and secretory function of the ependyma cells in the choroid plexus.

In the learning and memory assays, only a low number of young and aged rats has been tested. This pilot study provides just a preliminary result, namely, that there was no significant difference in the cognitive performance of normal aged and young rats indicating just a low non-symptomatic cerebral function loss with healthy aging. The diminished spatial memory function has already been described in earlier studies (Harrison et al., 2009; Kennard and Harrison, 2014) for APP–PSEN1 mice, which is a well-characterized model of Alzheimer’s disease.

Based on our cytokine array results, SCF, MCP-1, and RANTES can be further studied whether they can serve as biomarkers of aging measured from the plasma. Also, VEGF, PDGF-BB, and IL-17A can be proposed as markers of hypertriglyceridemia with brain dysfunction, and GM-CSF, IL-17A, and resistin can be indicators of Alzheimer’s-like neurodegeneration. To study the kinetics of the overexpression of these proteins during the progression of the pathology and the detectability from peripheral samples, further longitudinal experiments are needed.

In conclusion, the current study revealed the cerebral upregulation of VEGF, PDGF-BB, and IL-17A cytokines in APOB-100 mice and resistin, GM-CSF, and IL-17A induction in APP–PSEN1 transgenic mice, which indicates the possible role of these proteins in the Alzheimer’s-like pathology. The lack of BBB function in the nasal drug absorption in transgenic mice and also the unchanged cognitive status with healthy aging in normal rats have been shown. The brain imaging by MRI confirmed the previous data on enlarged cerebral ventricles in the APOB-100 mice (which can be the consequence of damaged energy metabolism and ependymal dysfunction) and lack of morphological abnormalities in APP–PSEN1 mice. Further studies are needed to analyze the effect of possible therapeutic interventions on the inflammatory balance shift that accompanies physiological and pathological aging processes.

## Data Availability Statement

The raw data supporting the conclusions of this article will be made available by the authors, without undue reservation.

## Ethics Statement

The animal study was reviewed and approved by the Directorate for the Safety of the Food Chain and Animal Health, Budapest and Pest County Agricultural Administrative Authority, Hungary.

## Author Contributions

ZV-M: nasal barrier studies, cytokine ELISA, data analysis, and manuscript editing. IH and DM: MRI studies and proofreading. MT and MS: APO-B-100 and APP–PSEN1 mice breeding, manuscript writing, and conceptualization. LB: cytokine ELISA and nasal experiments. KF: cytokine ELISA and nasal experiments. TI and PS: LC–MS/MS for determination of dialysate quinidine content. FE: conceptualization, grant application, manuscript writing, data analysis, and behavioral studies. All authors contributed to the article and approved the submitted version.

## Conflict of Interest

The authors declare that the research was conducted in the absence of any commercial or financial relationships that could be construed as a potential conflict of interest.

## Publisher’s Note

All claims expressed in this article are solely those of the authors and do not necessarily represent those of their affiliated organizations, or those of the publisher, the editors and the reviewers. Any product that may be evaluated in this article, or claim that may be made by its manufacturer, is not guaranteed or endorsed by the publisher.
